# Cardiac function and extracellular matrix morphology are altered by chronic high fat diet in *Drosophila* larvae

**DOI:** 10.1371/journal.pone.0330487

**Published:** 2025-08-22

**Authors:** Rachel M. Andrews, Saumya Naik, Katie Pelletier, J. Roger Jacobs

**Affiliations:** 1 Department of Biology, McMaster University, Hamilton, Canada; 2 Department of Zoology, University of British Columbia, Vancouver, Canada; 3 Lady Davis Institute for Medical Research, Montréal, Canada; 4 Division of Clinical and Translational Research, Faculty of Medicine, McGill University, Montréal, Canada; 5 Institut de Biologie de l’École Normale Supérieure, CNRS, Paris, France; BSRC Alexander Fleming: Biomedical Sciences Research Center Alexander Fleming, GREECE

## Abstract

Cardiovascular disease is characterized by aberrant and excessive extracellular matrix (ECM) remodelling, termed fibrosis. Fibrotic remodelling is typically triggered by inflammation, which occurs systemically in obesity. Despite the contribution of fibrosis to adverse clinical outcomes and disease progression, there are no available treatments for this condition. Developing therapeutics for chronic conditions requires an understanding of *in vivo* ECM regulation, and how the ECM responds to a systemic challenge. We have therefore developed a *Drosophila* model for obesity via chronic high fat diet feeding of larvae and evaluated the response of the cardiac ECM to this metabolic challenge. We found that this model displays a striking reorganisation of the cardiac ECM, with fibres oriented anterior to posterior, rather than in a complex network, suggesting tension modulation is altered. We also observe corresponding deficits in heart function, with high fat diet treatments resulting in an inability to contract the heart effectively at systole. Our study reveals that different genotypes tolerate different levels of dietary fat, and that some genotypes may require a different dietary supplementation regime to generate a cardiac phenotype. In summary, the *Drosophila* model for chronic high fat diet recapitulates many of the defects observed in human cardiovascular disease, allowing further evaluation of genetic and environmental influences on cardiac structure and physiology in disease states.

## Introduction

Cardiovascular disease (CVD) is a leading cause of death world-wide. In recent years, there has been an increase in age-adjusted mortality resulting from CVD [1]. Obesity is one of the main risk factors for the development of CVD, and an increased incidence of obesity has led to a corresponding increase in CVD rates [2]. An aspect of CVD that is often overlooked is the contribution of the extracellular matrix (ECM). The ECM is a protein scaffold that surrounds tissues within the body and acts to support their function by modulation of tension, distribution of forces through the tissue, sequestration of growth factors, and, relevant to the heart, mediation of electrical conduction [3–5]. The importance of the ECM is demonstrated by its intimate link to a variety of disease states, including cancer and CVD.

The ECM is composed of two main compartments, the interstitial matrix and the basement membrane. The interstitial matrix is composed primarily of fibrillar Collagen and forms a support scaffold, while the basement membrane is found close to the cell surface and acts as a barrier to the surroundings [4,6]. The ECM is not a static structure, and undergoes constant turnover, called remodelling. This process is a finely-tuned balance of matrix synthesis and deposition, as well as matrix breakdown [5,7]. The matrix metalloproteinases (MMPs) are mainly responsible for ECM breakdown, and their level of activity in the tissue is regulated by their inhibitors, the tissue inhibitors of metalloproteinases (TIMPs) [8]. The ratio of matrix deposition to breakdown is an important contributor to the biophysical properties of a tissue [5,9]. The levels of both MMPs and TIMPs are known to be altered in a variety of disease states, including diabetes and cancer [10,11].

Due to its critical involvement in supporting and maintaining tissue function, ECM homeostasis is tightly controlled [4]. One of the results of ECM dysregulation is fibrotic remodelling, or fibrosis. Fibrosis refers to increased deposition of matrix components, as well as increased levels of crosslinking between these proteins [3,12]. Increased levels of crosslinking insolubilize the matrix and make it more resistant to degradation, further disrupting the normal balance of remodelling [13].

In the heart specifically, fibrosis can have catastrophic consequences due to the replacement of highly specialized, contractile cardiomyocytes with non-contractile ECM proteins. This compromises the ability of the heart to contract effectively by altering the elastic properties of the tissue, disrupts the connections between cells that are crucial for conduction of nerve impulses, and leads to maladaptive cardiac remodelling. Overall, this reduces cardiac output and can progress to heart failure [3].

Despite being a prevalent component of many diseases, fibrosis has no available treatments [14]. In cases of CVD specifically, virtually every patient will exhibit some level of fibrotic remodelling [3,15]. Fibrosis of the heart is also known to occur in the context of obesity, one of the leading comorbidities of heart disease [16]. However, the development and progression of fibrosis as a result of obesity specifically is relatively uncharacterized. In order to design treatments that target the consequences of obesity itself, it is necessary to understand how the cardiac ECM is responding in this context.

We have employed *Drosophila melanogaster* to examine how obesity affects the cardiac ECM*. Drosophila* is a powerful tool for performing this research due to its lack of genetic redundancy, a simple heart tube that is not required to support larval life, and a similar basement membrane composition to mammals [7,17,18]. The *Drosophila* heart, or dorsal vessel, is a linear, tube-like structure that follows the same developmental pathways as the early mammalian heart. The *Drosophila* cardiac ECM is composed of the same core components as other basement membranes. It contains both Laminin and Collagen-IV, as well as the heart-specific collagen Pericardin. Pericardin is a Collagen-IV-like protein that organizes similarly to fibrillar collagens in mammals and has previously been shown to be critical for the maintenance of the *Drosophila* heart [19–21].

Diet has been used previously to induce obesity-like phenotypes in *Drosophila,* including methods that supplement a standard *Drosophila* diet with coconut oil as a source of fat and excess calories [22–24]. The present study utilized a dosage series of high fat diets (HFDs) to determine what affect dietary supplementation had on the growing *Drosophila* heart. Previous studies in mature adult *Drosophila* have revealed cardiac dysfunction, increased triglyceride levels, and altered metabolism as a result of short-term HFD feeding [22,23,25]. The present study aimed to better approximate chronic fat consumption like that seen in humans by feeding larval *Drosophila* a HFD from hatching. Larvae were allowed to feed until late third instar in order to maximize the duration of dietary treatment, generating a chronic high fat diet model. In *Drosophila,* growth and maturation are distinct stages, with growth occurring exclusively during larval stages [26]. By performing these experiments during the growth phase of the life cycle we are able to administer the HFD chronically, circumvent egg-laying and other adult behaviours, and maximally stress the heart as it must grow while adapting to HFD conditions. Utilizing larval stages also eliminates the confound of aging, which naturally leads to ECM accumulation [20,27]. Additionally, studies conducted on adult *Drosophila* heart function typically examine only females due to their larger body size [22,25,28]. Focusing on the larval life stage allows for analysis of both female and male larvae as size dimorphism is more limited during the growth stage. This allows us to examine any sex-specific effects of HFD treatments more easily than in adult *Drosophila*.

Additionally, a high sucrose diet was employed in this study to determine if the type of nutrient providing the excess calories affected the heart specifically. High sucrose diets have been shown to induce a diabetes-like phenotype in *Drosophila*, exhibiting insulin resistance, cardiac arrhythmias, and accumulation of Pericardin [29]. This dietary treatment allows for a distinction to be made between excess caloric intake or inducing an obesity phenotype as the cause of cardiac dysfunction.

Here, we describe the effects of chronic HFD feeding on the larval *Drosophila* cardiac ECM. We observed that HFD feeding results in several hallmarks of obesity and causes changes to matrix organization of both Pericardin and Collagen-IV. Pericardin organization was severely perturbed, with the protein network exhibiting an anterior-posterior fibre alignment phenotype that was rarely observed in controls. The Collagen-IV matrix demonstrated a clumping phenotype, with a dose-dependent level of clumping within the matrix. We also observed functional impairment of the heart, namely an inability to contract fully at systole. This could be due to the rearrangement of the cardiac ECM and altered tension through the dorsal vessel. Overall, our results suggest that chronic HFD feeding in *Drosophila* larvae induces an obesity-like phenotype that affects the organization of the cardiac ECM and leads to cardiovascular impairment.

## Results

### Larvae fed high fat and high sucrose diets display obesity-like phenotypes

To establish a dilution series of HFD treatments, larvae were reared from hatching on food containing 10%−50% coconut oil. Protein concentration was constant in all treatments. At 50% coconut oil supplementation no larvae survived to late L3, so 10%−40% dosages were used for all experiments. No significant developmental delay was observed with HFD treatment. 1M sucrose (high sucrose diet) was selected based on previous studies [30] and is calorically comparable to 20% HFD supplementation. Larvae did not survive on a 5M sucrose diet, which would have been equivalent in calories to the 40% HFD treatment. All assays performed separated female and male larvae in order to isolate possible sex specific effects. Assays were also conducted on both *y*^*1*^*w*^*1118*^ and *y*^*1*^*w*^*1118*^*; vkg-GFP (vkg*^*CC00791*^, hereafter *vkgGFP*) so both Collagens, Pericardin and *viking*, in the cardiac ECM could be examined. *VkgGFP* is a protein trap that labels the α2 subunit of Collagen-IV with a GFP tag, so endogenous Collagen-IV is fluorescently tagged [31].

**Fig 1:**
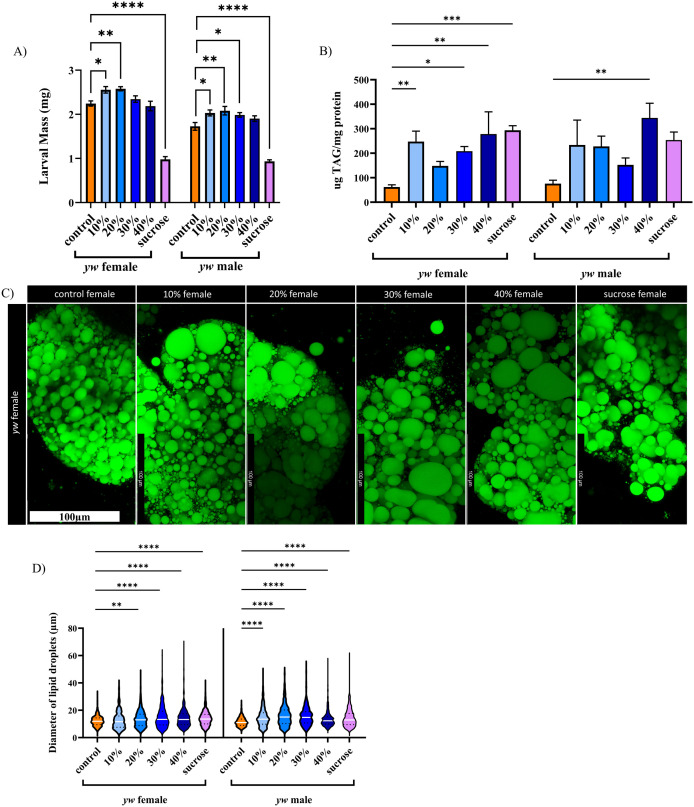
*y*^*1*^*w*^*1118*^ larvae fed a high fat diet display a dose dependent increase in markers of obesity. Larval mass is not lowered by high fat diet supplementation, in contrast to high sucrose supplementation (A). HFD feeding results in a dose-dependent increase in triglyceride levels in *y*^*1*^*w*^*1118*^ larvae, especially females (B). Lipid droplets were visualized with BODIPY 493/503 (C) and both HFD feeding and a high sucrose diet result in a dose-dependent increase in lipid droplet size (D). Error bars in A and B are SEM. Scale bar in C is 100µm. White lines in D represent the median, dotted lines represent quartiles. n > 10 individuals for all groups. * = p < 0.05, ** = p < 0.01, *** = p < 0.001, **** = p < 0.0001. If no p value is indicated, comparison is not statistically significant.

The health of larvae was assessed by measuring larval mass, triglyceride levels, and lipid droplet diameter. In *y*^*1*^*w*^*1118*^ high fat diet treatment groups, larval mass was mildly but significantly elevated in 10% and 20% supplemented females, and 10%−30% supplemented males (elevation between 13.8–14.8% in females, and 15.1–20.6% in males) (Fig 1A). A modest increase or unchanged body mass was not unexpected as the critical weight checkpoint triggers the transition from larval development to pupation [32]. An increase in mass could therefore trigger early pupation rather than continued increase in larval mass. The high sucrose diet resulted in larvae that were significantly smaller than controls and the calorically equivalent HFD treatment (females 43.6% mass of controls, males 54%) (Fig 1A). This is consistent with previous studies that demonstrate high sucrose diets induce diabetes-like phenotypes as a result of impaired insulin signalling [29]. Triglyceride levels were quantified and *y*^*1*^*w*^*1118*^ HFD fed larvae exhibited a dose-dependent increase in triglyceride levels compared to controls (Fig 1B). Both female and male larvae showed an increase, with female larvae exhibiting a more significant change. Lipid droplet diameter from the larval fat body in *y*^*1*^*w*^*1118*^ individuals exhibited a dose dependent effect with HFD feeding (Fig 1C). All *y*^*1*^*w*^*1118*^ HFDs had increased lipid droplet diameter, except 10% HFD females (Fig 1D, S1 Fig). The high sucrose diet also exhibited significantly increased droplet diameter. Unfortunately, the BODIPY 493/503 (green) dye was indistinguishable from background *vkgGFP* fluorescence and BOPIDY 630/650 (red) dye binds to cell membranes instead of entering the lipid droplet, so lipid droplet diameter could not be quantified in this genotype.

**Fig 2:**
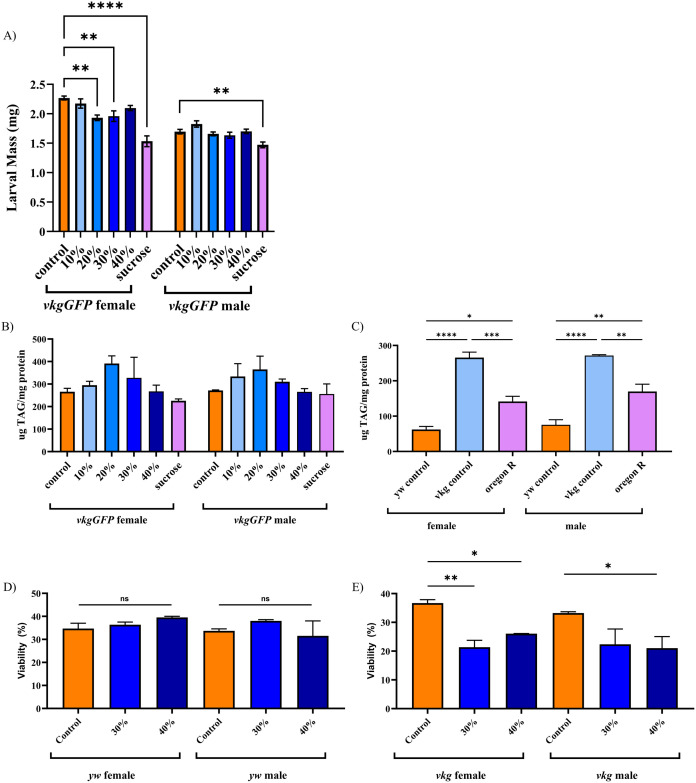
*vkgGFP* larvae have elevated baseline triglyceride levels and do not tolerate HFD supplementation as well as *y*^*1*^*w*^*1118*^. *vkgGFP* larval mass is lower in 20% and 30% HFD fed females and unchanged in other HFD supplementation groups (A). *vkgGFP* larvae did not have significantly elevated triglyceride levels in response to HFD feeding (B), but control individuals were found to possess markedly elevated baseline triglyceride levels compared to *y*^*1*^*w*^*1118*^ larvae (C). Larvae of the genotype Oregon R were found to have intermediate triglyceride levels to *vkgGFP* and *y*^*1*^*w*^*111*^ (C). HFD supplementation was not found to affect the viability of *y*^*1*^*w*^*1118*^ larvae (D), but did result in reduced viability of *vkgGFP* larvae (E). Error bars are SEM. * = p < 0.05, ** = p < 0.01, *** = p < 0.001, **** = p < 0.0001. If no p value is indicated, comparison is not statistically significant.

In *vkgGFP* larvae 20% and 30% HFD females were smaller than the *vkgGFP* larvae fed a control diet. This suggests that *vkgGFP* does not tolerate the HFD treatment as well as the *y*^*1*^*w*^*1118*^ genotype (Fig 2A). *vkgGFP* larvae did not demonstrate a statistically significant elevation in triglyceride level (Fig 2B) but *vkgGFP* control-diet fed larvae had triglyceride levels over 4 times higher than *y*^*1*^*w*^*1118*^ larvae fed a control diet (Fig 2C). An additional wildtype strain, Oregon R, had triglyceride levels intermediate between *y*^*1*^*w*^*1118*^ and *vkgGFP,* suggesting that triglyceride levels can vary markedly with genotype (Fig 2C). The higher baseline triglyceride level in *vkgGFP* individuals may contribute to their reduced ability to tolerate HFD feeding when compared to *y*^*1*^*w*^*1118*^*.* Survival data for 30% and 40% supplemented diets did not reveal altered viability for the *y*^*1*^*w*^*1118*^ genotype (Fig 2D), but significantly reduced viability in *vkgGFP* larvae fed the same diets (Fig 2E). This indicates an inability of the *vkgGFP* genotype to tolerate higher dietary supplementation of fat. It also suggests that there is a limit to the level of triglycerides that larvae are able to process effectively.

Overall, this demonstrates that a chronic high fat diet induces obesity-like phenotypes, while high sucrose diets have some characteristics of obesity but are significantly smaller than controls and HFD treatments. Previous studies have shown that this is due to changes in insulin signalling that induces a diabetes-like phenotype in these individuals [30].

### Pericardin fibre organization is altered with HFD treatment

**Fig 3:**
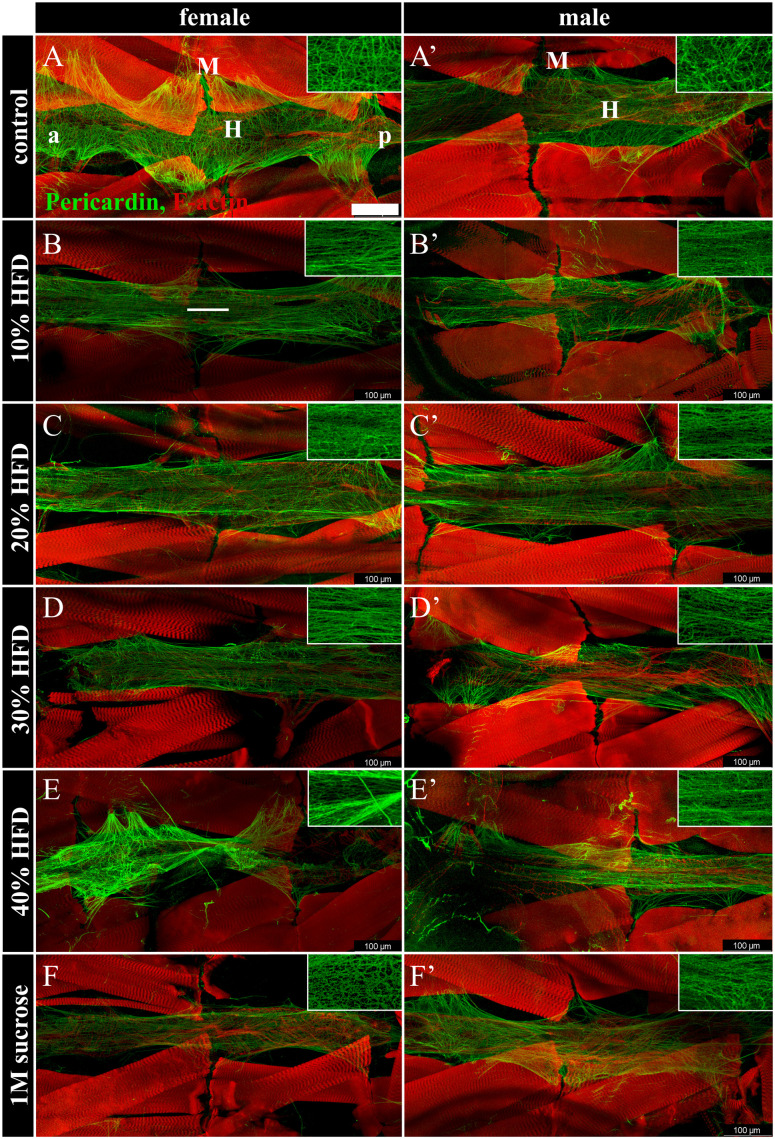
Pericardin fibre organization is perturbed in *y*^*1*^*w*^*1118*^ dietary treatments. Controls demonstrate a normal, organized meshwork (A-A’) while dietary treatments exhibit a change in matrix organization, with matrix fibres becoming oriented anterior-posterior (B-F’). The cardiac ECM is visualized by immunolabelling Pericardin (green) and F-actin (red). The F-actin label in the background is body wall muscle. In panel A, H labels the heart tube, M labels alary muscles, a is anterior, p is posterior. All images are oriented anterior to the left. Pericardin insets are enlarged approximately 2.5x. Scale bar in A is 100µm. White line in panel B highlights the anterior-posterior orientation of Pericardin fibres. n > 10 for all groups.

**Fig 4:**
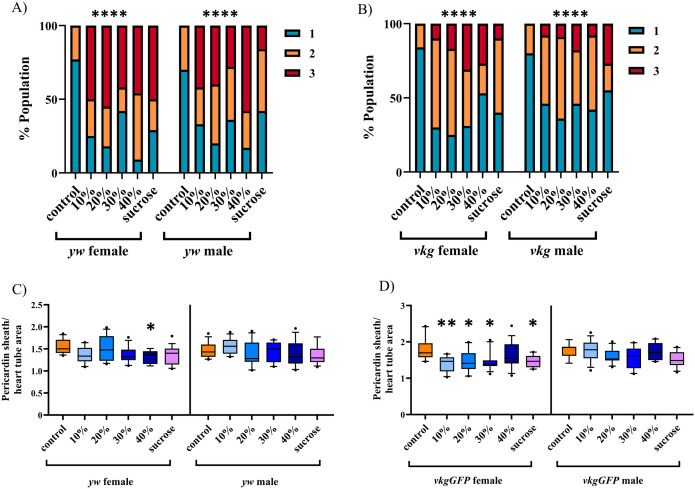
Matrix organization reveals severe rearrangement in dietary treatments. Matrix organization was scored on a scale of 1 (normal matrix organization) to 3 (the majority of matrix organization is disrupted). Dietary treatments demonstrate a linearity phenotype that is more common and more severe than controls in both *y*^*1*^*w*^*1118*^ (A) and *vkgGFP* larvae (B). In females, there is a downward trend in the size of the Pericardin matrix relative to the heart tube in *y*^*1*^*w*^*1118*^ (C) and *vkgGFP* (D). Males of all genotypes fail to reveal a significant trend. n > 10 for all groups. Panels A and B use a Chi squared test, figures C and D a one-way ANOVA with Dunnett’s correction. * = p < 0.05, ** = p < 0.01, **** = p < 0.0001. If no p value is indicated, comparison is not statistically significant.

**Fig 5:**
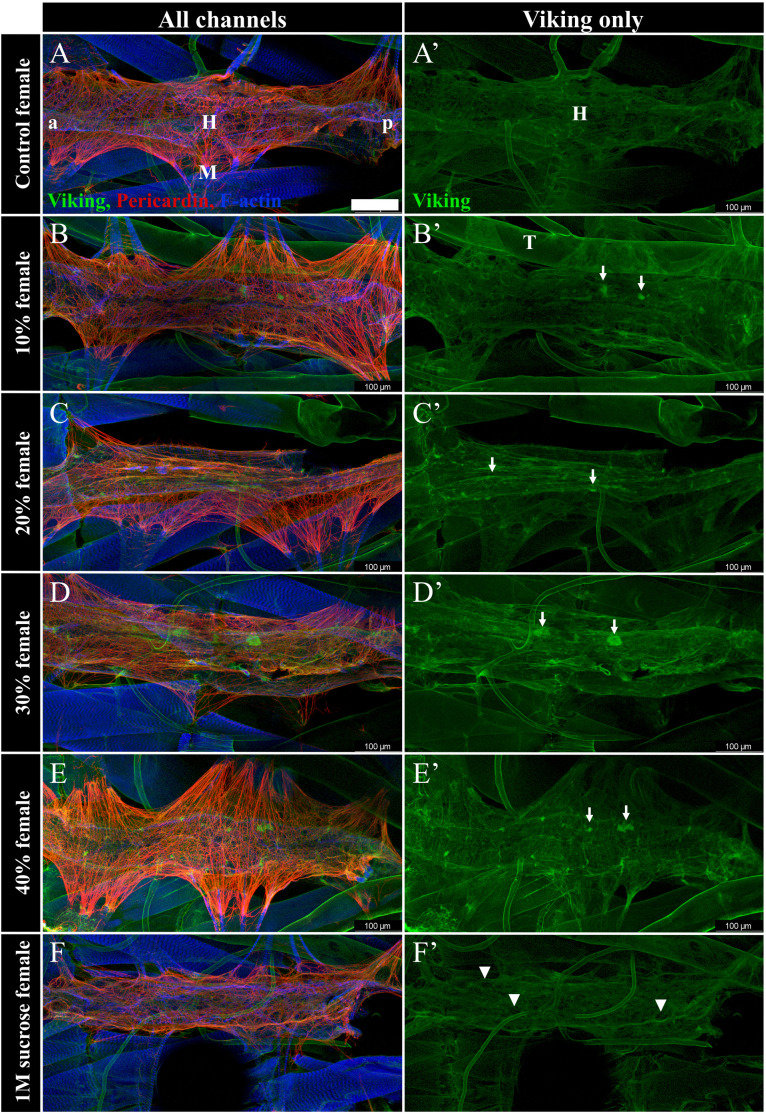
Collagen-IV distribution is abnormal in *vkgGFP* dietary treatments. In control individuals the Collagen-IV matrix possesses a uniform, sheet-like distribution across the surface of the heart (A’). HFD treated larvae exhibit elevated levels of clumping within the Collagen-IV matrix, indicated by white arrows, that is not found in the high sucrose diet treatment (B’, C’, D’, E’, F’). Gaps are observed in the high sucrose Collagen-IV matrix, indicated by white arrowheads (F’). The cardiac ECM is visualized by endogenous *vkgGFP* fluorescence (green), and immunolabelling of Pericardin (red) and F-actin (blue). In panel A, H labels the heart tube, M labels alary muscles, a is anterior, p is posterior. *vkgGFP* is also expressed in the trachea, indicated by T in panel B’. All images are oriented anterior to the left. Scale bar in A is 100µm. n > 10 for all groups.

Having established that HFD feeding results in several hallmarks of obesity, we investigated the effect of a chronic HFD on the cardiac ECM. The fibrous, heart specific, collagen-like Pericardin revealed marked changes in its organization with all dietary treatments. A normal Pericardin matrix is an organized meshwork that forms a honeycomb-like pattern and extends away from the heart tube, connecting it with heart associated nephrocytes and alary muscles that suspend the heart below the dorsal surface (Fig 3A, A’). With dietary treatments, the matrix takes on an anterior-posterior linearity phenotype (Fig 3B-F’) that is rarely observed in controls. This phenotype was blind scored by two independent individuals using the following criteria: a scale of 1–3, with 1 representing a normally organized matrix, 2 representing a matrix with between 10 and 49% linear fibres, and 3 representing a matrix where the majority displays a linear matrix organization. Both *y*^*1*^*w*^*1118*^ and *vkgGFP* larvae (matrix organization shown in Fig 5A-F) exhibit this phenotype in response to all dietary treatments (Fig 4A & B). Interestingly, in *vkgGFP* specifically, there is a slight improvement in the percentage of the population that is affected at 40% feeding (Fig 4B). This was likely due to a survivor bias in this group, as survival of *vkgGFP* larvae was significantly reduced at a 40% HFD (Fig 2E). This suggests that HFD treatments that lead to mortality of the larvae correlate with the most significantly disrupted ECMs in *vkgGFP* larvae, indicating that *vkgGFP* appears to be less able to tolerate the highest HFD treatments than *y*^*1*^*w*^*1118*^ larvae.

The Pericardin matrix also appears to not extend away from the heart tube laterally with both high fat and high sucrose diets (Figs 3B-F’, 5B-F’). This could be due to altered tension due to changes in matrix organization. The ratio of the area of the Pericardin matrix to the area of the heart tube revealed a dose-dependent downward trend in female *vkgGFP* larvae (Fig 4D). A similar trend was observed in the female *y*^*1*^*w*^*1118*^ larvae but was not statistically significant until 40% fat supplementation was reached (Fig 4C). This trend was not observed in males of either genotype.

### Collagen-IV distribution is affected by dietary treatments

**Fig 6:**
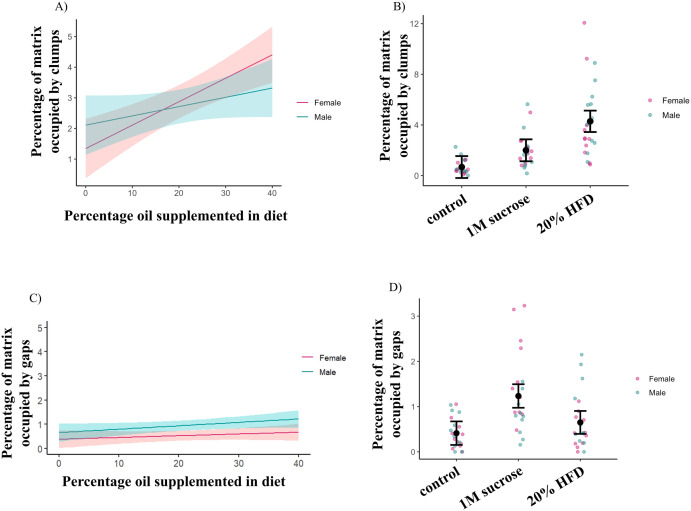
Collagen-IV clumping depends on dosage of HFD treatment. HFD feeding results in a dose-dependent increase in the amount of clumping within the Collagen-IV matrix (p < 0.0001) (A). High sucrose supplementation did not demonstrate increased Collagen-IV clumping compared to controls while the calorically comparable HFD does (CI = control −0.190896–1.5403, sucrose 1.13279–2.864116, 20% HFD 3.4414–5.131001) (B). The Collagen-IV matrix does not possess more gaps with HFD supplementation (p > 0.05) (C). High sucrose supplementation results in a Collagen-IV matrix with more gaps when compared to both controls diets and the calorically equivalent HFD treatment (CI = control 0.153–0.673, sucrose 0.974–1.494, 20% HFD 0.396–0.904) (D). Graphs in A and C are a model estimate, shown by the solid line, with 95% confidence intervals, indicated by the shaded area. In both A and C 0% supplementation refers to the control diet. Error bars in B and D represent 95% confidence intervals. n > 10 for all groups.

The Collagen-IV matrix was visualized using the *vkgGFP* line. The normal distribution of this matrix is sheet-like, covering the entire surface of the heart (Fig 5A’). When observing the response of the Collagen-IV matrix to dietary treatments, clumps of Collagen-IV were visible (Fig 5B’-E’). The area of the clumps was quantified as a percentage of the total Collagen-IV matrix area in all treatment groups. Control larvae were found to possess a matrix with approximately 0.67% (95% CI: 0–1.5%) of the Collagen-IV matrix occupied by clumps. HFD treatment groups demonstrate a dose-dependent increase in levels of Collagen-IV clumping (Fig 6A). When comparing a calorically equivalent 20% HFD to the high sucrose supplementation group, the high sucrose group was not found to have elevated clumping levels (Fig 6B).

While clumping was not observed in the high sucrose dietary treatment, this group did have an increased area occupied by gaps in the normally sheet-like matrix compared to a control diet (1.32% increase, 95% CI: 2.79, 0.15) (Fig 6D, ECM images in 5F, F’). The high fat diet treatment groups did not exhibit this phenotype (Fig 6C). This suggests that the defects in Collagen-IV deposition and remodelling may vary with treatment type, perhaps due to reduced growth and altered physiology in the high sucrose group.

### HFD treatment impairs ability of heart to contract at systole

**Fig 7:**
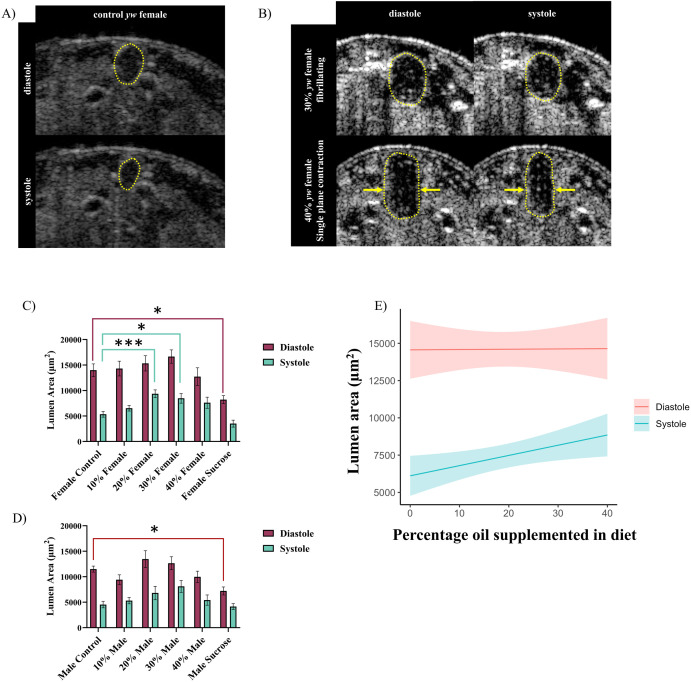
Optical coherence tomography reveals impaired contraction with HFD treatment. OCT was used to visualize the heart beating in cross-section, revealing the area inside the lumen at both diastole and systole (A). Control hearts are round or oval and contract evenly along the perimeter, while HFD treatment hearts are abnormally shaped and unable to contract evenly on all sides (B). Lumen cross-sectional area in *y*^*1*^*w*^*1118*^ females displays a change in diastole only in high sucrose individuals (C), but does reveal increases in systolic area at higher concentrations of HFD. A similar trend is observed in males (D). HFDs demonstrate a dose-dependent impairment of heart contractions (E). Yellow outlines in A and B trace the heart lumen. Yellow arrows in B indicate plane of contraction. Error bars in C and D are SEM. Graph in E is a model estimate, shown by the solid line, with 95% confidence intervals, indicated by the shaded areas. n > 10 for all groups. * = p < 0.05, ** = p < 0.01, *** = p < 0.001, **** = p < 0.0001. In panels C and D, if no p value is indicated the comparison was not statistically significant.

If the cardiac ECM of larvae raised on a HFD reveal perturbations in the organization of both Viking and Pericardin, how then might this affect cardiac physiology? We performed live imaging to determine if these ECM perturbations have functional consequences using optical coherence tomography (OCT). Movies of beating hearts (see supplemental movies) were used to measure the cross-sectional area of the lumen at both diastole and systole (Fig 7A), and it was found that the high sucrose diet generates larvae with much smaller hearts than larvae fed a control diet (Fig 7C, D). This is likely due to the smaller body mass of these individuals compared to their control counterparts. The HFD treatments had similar diastolic areas across all treatment groups when compared to the control diet. However, the high fat diet demonstrated a dose-dependent increase in systolic area (Fig 7E). This indicates an inability of the heart to contract fully with increasing dietary fat supplementation, suggesting contractility may be impaired by reduced elasticity of the ECM, similar to observations from both human data and mammalian CVD models [33,34]. However, heart rate was unaffected (S2 Fig). Rhythmicity was examined by calculating an arrhythmicity index and this was found to be elevated only in *y*^*1*^*w*^*1118*^ high sucrose diet males (S2 Fig). Live imaging also revealed that the hearts of HFD treated larvae were more likely to be abnormally shaped (Fig 7B). The majority of control diet fed larvae possessed hearts that were close to round or oval in cross section and contracted evenly on all sides. In the HFD treated individuals there were irregularly shaped hearts (Fig 7B), as well as contraction that mainly occurred in one plane instead of uniformly around the circumference of the heart (see supplemental movies).

## Discussion

A chronic high fat diet in growing *Drosophila* larvae generates dose-dependent effects on overall organismal health as well as on the form and function of the heart. HFD individuals show clear signs of obesity and CVD, while the high sucrose diet shares some markers of obesity as well as other metabolic disorders. Previous studies in adult *Drosophila* have found that transient HFD feeding leads to increased fat storage, including ectopic deposition of triglycerides, as well as cardiac dysfunction, including decreased diastolic and systolic diameters, reduced fractional shortening (a proxy for stroke volume), and reduced heart period [25,35]. These health effects point towards the development of lipotoxic cardiomyopathy. Here, we find that growing larval *Drosophila* experience similar whole-body symptoms, including increased triglyceride levels and altered lipid droplet morphology, but that an inability to contract at systole was the main functional parameter affected by chronic HFD in larvae. No reductions in diastolic area or heart rate were observed. Additionally, we observed changes to the organization of the cardiac ECM that may suggest altered ECM dynamics play a role in observed functional defects.

Genotype was found to affect the sensitivity of larvae to chronic HFD feeding. *y*^*1*^*w*^*1118*^ individuals tolerated the HFD more readily than *vkgGFP* individuals, despite the *vkgGFP* line being derived from the same genetic background. This may be due to the elevated triglyceride levels found in *vkgGFP* control larvae. These larvae have comparable triglyceride levels to 40% HFD fed *y*^*1*^*w*^*1118*^ individuals, suggesting that they may be close to a maximum level of tolerance of triglycerides. The increase due to HFD feeding may have caused the reduced viability seen in *vkgGFP* in contrast to *y*^*1*^*w*^*1118*^ fed a high fat diet. Oregon R larvae fed a control diet possess triglyceride levels intermediate to *y*^*1*^*w*^*1118*^ and *vkgGFP,* suggesting that triglyceride levels on ordinary lab diets are a variable trait.

HFD feeding affected cardiac ECM organization, with both the Pericardin and Collagen-IV matrices displaying defects. Collagen-IV phenotypes for HFD and high sucrose diets were different, with HFD treatments exhibiting a clumping phenotype while high sucrose diets possessed gaps within the matrix. The Collagen-IV clumping phenotype in the HFD feeding treatments suggest that matrix deposition may be elevated. This could be due to increased expression of Collagen-IV or to reduced breakdown (or turnover) of the matrix. Matrix breakdown is performed by the matrix metalloproteinases (MMPs) and altered levels or activity of these proteins have been shown to promote fibrotic remodelling [36,37]. Altered MMP expression profiles have been demonstrated in a variety of fibrotic diseases [38,39]. We described previously how depletion of MMP2 by overexpression of its inhibitor TIMP during larval growth causes accumulation of Collagen-IV, suggesting MMP2 expression is required for the maintenance of a healthy ECM [8]. Reduced MMP2 activity, either as a result of altered gene expression or due to elevated expression of TIMP, presents an intriguing candidate for the clumping observed in the Collagen-IV matrix in these HFD treatment groups.

Organization of the Pericardin network was severely affected by HFD feeding. Instead of forming a honey-comb like meshwork as in larvae fed a control diet, HFD feeding and the high sucrose diet exhibited an anterior-posterior fibre linearity. This linearity phenotype was observed in all HFD treatments. The typical arrangement of the ECM has Pericardin fibres extending in many directions in order to transmit tension evenly across the heart. This allows for the heart to open evenly in all directions and helps to maintain a uniform shape as the heart contracts and relaxes. Increased linearity of fibres in HFD treated larvae correlates with defects in the ability of the heart to contract evenly around its perimeter. Both effects suggest that HFD feeding results in an uneven distribution of tension around the heart by affecting the organization of the ECM. Additionally, live imaging by OCT revealed a dose-dependent inability of the heart to contract fully at systole. In contrast to studies performed in adult *Drosophila,* diastolic area is unaffected, indicating the defect observed here is in the ability of the heart to contract, not relax. A common finding in CVD in humans is an inability of the heart to contract effectively, including in cases of obesity-related CVD [40]. Pericardin linearity is rarely seen in controls, suggesting that the ability to organize the matrix appropriately is affected by HFD feeding. This could be due to increased inflammatory responses in response to a high fat diet. Excess triglycerides are known to cause an inflammatory response which can promote fibrotic remodelling of fibrillar Collagens in cases of CVD in mammalian systems [16,41]. The connection between matrix organization and cardiac function shown here demonstrates that the amount of extracellular matrix proteins, for example collagens, is not the only determining factor in the severity of functional defects. Our results suggest that further study can focus on the importance of matrix organization in the development of cardiovascular disease.

Additionally, ECM regulation is known to be affected in disease states, with elevated levels of crosslinking enzymes, altered post-secretion activation of MMPs and TIMPs, and alterations to proteins important for appropriate Collagen deposition like its chaperone, SPARC [7,42–44]. Both MMP and SPARC have previously been shown to be upregulated in heart disease, show changes in expression as a result of inflammation, and have been implicated in fibrotic remodelling of Collagen [43,44]. Our previous study also found that ongoing MMP activity is critical for the appropriate organization of the growing Collagen-IV matrix [8]. It is therefore feasible that Pericardin organization requires specific post-translational modifications or enzymatic activity in order to form a normally organized network. The observed changes in the Pericardin matrix could reflect changes in MMP and SPARC function that result from chronic inflammation, thereby generating a matrix with less structural integrity.

*Drosophila* fed a HFD exhibit many of the same metabolic and physical symptoms as obese humans [22,25,35]. However, existing studies have predominantly focused on transient feeding of adults, and analyzed only females. Our work suggests that larval *Drosophila* provides an effective model for chronic dietary treatments, and for the examination of the sex specific effects of diet. We also find that while previous studies have reported most consistent and reproducible effects of HFD treatments on 30% coconut oil supplemented diets [22,23,25], there may be variability in tolerance between genotypes. Our results reveal that 30% supplementation is ideal for inducing cardiac phenotypes in *y*^*1*^*w*^*1118*^*,* but that a 20% diet is better for *vkgGFP.* This presents an interesting opportunity to examine the effects of genetic background on response to dietary treatment.

It has previously been found that a HFD affects heart function due to increased TOR signalling and decreased expression of the lipase Brummer [22]. TOR signalling is highly conserved and also altered in human disease. Based on our findings it is possible that different *Drosophila* genotypes have different activity levels of key pathways, thereby altering their ability to respond to the lipotoxic insult of a HFD. Further study can utilize this feeding regime as a model for understanding the genetic basis of differing tolerances to HFD feeding, which may provide insight into how these processes are differentially regulated in human populations.

## Methods

### *Drosophila* strains and dietary treatments

*y*^*1*^*w*^*1118*^ (6598, BDSC) and *y*^*1*^*w*^*1118*^*; vkg-GFP (vkg*^*CC00791*^*)* lines were used for these experiments [31] (Bloomington stock centre, NIH P40OD018537). Flies were maintained at room temperature. Larvae were fed one of six different dietary treatments from hatching – regular fly food (control), 1.0M sucrose, 10%, 20%, 30%, and 40% coconut oil supplemented (high fat diet). In all dietary treatments the protein source was scaled to match the volume of food. 1.0M sucrose is approximately calorically equivalent to 20% HFD. All experiments were performed using wandering third instar larvae.

All treatments were supplements made to standard lab food, which consists of 3.6L of water, 300g sucrose (0.2M), 150g yeast, 24g KNa tartrate, 3g dipotassium hydrogen orthobasic, 1.5g NaCl, 1.5g CaCl_2,_ 1.5g MgCl_2_, 1.5g ferric sulfur, and 54g of agar. Fly food is autoclaved, cooled to 55^0^C, then 22mL of 10% tegosept and 15mL of acid mix is added before dispensing. Coconut oil supplements were by volume, sucrose by molarity.

### Viability

Embryos were laid on apple juice agar plates for 24 hours. Viability was quantified by transferring 100 embryos of the desired genotype onto a plate containing the appropriate food. Larvae were allowed to grow to the third larval instar and the surviving females and males were counted at this stage.

### Triglyceride assay

Triglyceride levels were measured using a serum triglyceride determination kit (Sigma Aldrich, TR0100) [45]. 5 intact third instar larvae (a replicate) were flash frozen in liquid nitrogen and stored at −80^0^C before sample preparation. A minimum of 3 replicates for each treatment group were measured. Frozen larvae were ground with a manual homogenizer in 0.1% Tween in PBS. 20µl of buffer per larva was used. Samples were heat treated at 70^0^C for 10 minutes, then centrifuged at maximum speed for 3 minutes. 10 µL of each replicate was loaded into a 96 well plate in triplicate. 10 µL of a glycerol standard at 2.5 mg/mL, 1.25 mg/mL, 0.625 mg/mL, 0.315 mg/mL, 0.156 mg/mL, and 0 mg/mL were also loaded. 250 µL of free glycerol reagent was added to each well, incubated at 37^0^C, and absorbance was read at 540nm. 50 µL of triglyceride reagent was then added, incubated for 10 minutes at 37^0^C, and absorbance read at 540nm. The change in glycerol levels after addition of the triglyceride reagent was calculated to determine the level of stored triglycerides in the sample. A Bradford assay was then conducted on the same samples, and the level of stored triglycerides was divided by the amount of protein in the sample to control for body size.

### Dissections

#### Heart.

Dissections were performed by fixing larvae dorsal down to a surface using pins [46]. Larvae were bathed in PBS and an incision was made at the ventral midline. The cuticle was pinned back, and the gut and fat bodies were removed to reveal the heart. Dissections were performed at third instar, after the onset of wandering behaviour.

#### Fat body.

Above process was followed but only the gut was removed to expose the fat bodies.

### Immunohistochemistry

#### Heart.

Relaxed hearts were fixed for 20 minutes without shaking at room temperature in 4% paraformaldehyde in PBS. Specimens were then washed 3x10 minutes in PBST (0.3% Triton-X-100), before blocking for 30 minutes with NGS (1:15). Primary antibodies were incubated overnight at 4°C with shaking. After incubation with primary 3x10 minute washes in PBST were performed before adding secondary antibodies for one hour at room temperature. Phalloidin was added at the same time as secondary antibodies. Specimens were then washed 3x10 minutes in PBST, with a final wash in PBS to remove detergent. 50% glycerol was added for at least 3 hours, then 70% glycerol overnight. The primary antibody used was mouse anti-Prc (Pericardin, EC11, DSHB, 1:30 dilution). Secondary antibodies used were Alexa Fluor 488 anti-mouse and Alexa Fluor 647 anti-mouse (1:150 dilution). Alexa Fluor 546 and 647 Phalloidin (Thermofisher Scientific) were also used (1:75 dilution).

#### Fat body.

Dissections were fixed for 30 minutes at room temperature in 4% paraformaldehyde. Specimens were washed 2x5 minutes in PBST, then incubated in 493/503 BODIPY (1:1000) for 30 minutes. Specimens were then washed 2x5 minutes, placed in 70% glycerol, and immediately mounted for imaging.

### Imaging

A Leica SP5 confocal microscope was used to obtain image stacks. 1µm intervals between frames were used for heart dissections, 0.5µm intervals were used for fat bodies. Fat bodies were imaged from the surface to a depth of 30µm. Hearts were imaged from the ventral face of the cardiac ECM to the dorsal edge of the heart tube, for segments A6-A7. Images were processed using Leica software (LAS AF), ImageJ, and ZEN blue.

### Image quantification and statistics

To assess Pericardin linearity, images were double blinded and scored, with a scale of 1 being a normal meshy matrix, 2 having some linearity but not the majority, and 3 being majority or completely linear.

Pericardin matrix to heart tube ratio was obtained by generating a 3D projection of the heart tube, then tracing the outline of the heart tube from the Phalloidin channel and the outline of the extracellular matrix from the Pericardin channel. An area was obtained for each, and a ratio was generated using these values.

The percentage of the Collagen-IV matrix that was occupied by clumps was obtained by tracing the total area of the matrix and then tracing the area of each clump within the matrix. The sum of clump areas was then expressed as a percentage of the total matrix area. Gaps were quantified in the same manner.

Lipid droplet diameter was measured using the line tool in ZEN blue [32]. All lipid droplets within the field of view were measured.

All measurements from confocal images were performed on unedited images. For publication only images have had brightness and colour balance adjusted using Photoshop CS6.

Statistical analysis of larval health (mass, triglyceride levels, lipid droplet size), Pericardin matrix size, and diastole/systole were performed using Graphpad Prism (v.9.5.1). Analysis of variance (ANOVA) with a Dunnett’s multiple comparisons test was performed. Graphs are plotted with SEM. A Chi squared test was performed to assess Pericardin linearity.

For Collagen-IV matrix organization statistics we fit a linear model with the terms sex, diet, and their interaction using the R programming language. Significance of the terms was tested with ANOVA and differences in group means using the emmeans(v1.7.2) package, with 95% confidence intervals reported for all statistics. Sucrose and the equivalent calorie HFD were compared to controls separately to determine if sucrose had a comparable effect to HFD feeding. Because sucrose was not different from controls it was excluded from HFD comparisons.

Diastolic and systolic volumes for HFD treatments were fit using a linear model with the term diet to estimate the slope of the line as 95% confidence intervals.

### OCT imaging

Optical coherence tomography (OCT) was used to visualize the heart beating *in vivo* in real time in late third instar larvae. Larvae were adhered to a microscope slide dorsal side up before being placed under the OCT camera. B scans were taken in 3D acquisition mode using a Thorlabs OCT Telesto series TEL221PS system at the widest point of the heart chamber with the following parameters: X size 1257 pixels, 1.03 mm, Y size 0, 400 frames, Z field of view 1.2 mm. This gives a 20 second video with 20 frames per second. Image stacks were then exported as TIFs and processed in ImageJ [47]. The cross-sectional area was measured at both diastole and systole. Diastolic and systolic volumes across HFD groups were compared using a model estimate with 95% confidence intervals. Rhythmicity was calculated by measuring the distance between diastolic peaks and generating a rhythmicity index [48].

## Supporting information

S1 Fig Female and male health parameters. Figs 1A, B, and D organized to show female and male data beside each other. Error bars are SEM. * = p < 0.05, ** = p < 0.01, *** = p < 0.001, **** = p < 0.0001. If no p value is indicated, comparison is not statistically significant.
(TIF)

S2 Fig Cardiac functional parameters are unchanged in most dietary treatments. Heart rate was not significantly different with any dietary treatment (A). Arrhythmicity index was unaffected in all treatments except for high sucrose diet males (B). Error bars are SEM. ** = p < 0.01.
(TIF)

S1 File Control heartbeat. Example of a *yw* female heart beat recorded using OCT. The heart contracts evenly around the perimeter, and reaches a small size at systole.
(AVI)

S2 File 20% HFD heartbeat. Example heartbeat of a *yw* female fed a 20% HFD. This individual displays a fibrillation defect where the heart does not contract fully with each beat.
(AVI)

S3 File 40% HFD heartbeat. Example heartbeat of a *yw* female fed a 40% HFD. This individual has a contraction defect where the heart only contracts in one plane instead of evenly around the entire perimeter.
(AVI)

S1 Data Raw data and statistics sheet. All raw data used in this study, and the statistical analyses performed.
(XLSX)

## References

[1] Sidney S, Lee C, Liu J, Khan SS, Lloyd-Jones DM, Rana JS. Age-Adjusted Mortality Rates and Age and Risk-Associated Contributions to Change in Heart Disease and Stroke Mortality, 2011-2019 and 2019-2020. JAMA Netw Open. 2022;5(3):e223872. doi: 10.1001/jamanetworkopen.2022.3872 35319764 PMC8943624

[2] Poirier P, Giles TD, Bray GA, Hong Y, Stern JS, Pi-Sunyer FX, et al. Obesity and cardiovascular disease: pathophysiology, evaluation, and effect of weight loss: an update of the 1997 American Heart Association Scientific Statement on Obesity and Heart Disease from the Obesity Committee of the Council on Nutrition, Physical Activity, and Metabolism. Circulation. 2006;113(6):898–918. doi: 10.1161/CIRCULATIONAHA.106.171016 16380542

[3] Travers JG, Kamal FA, Robbins J, Yutzey KE, Blaxall BC. Cardiac Fibrosis: The Fibroblast Awakens. Circ Res. 2016;118(6):1021–40. doi: 10.1161/CIRCRESAHA.115.306565 26987915 PMC4800485

[4] Bonnans C, Chou J, Werb Z. Remodelling the extracellular matrix in development and disease. Nat Rev Mol Cell Biol. 2014;15(12):786–801. doi: 10.1038/nrm3904 25415508 PMC4316204

[5] Cox TR, Erler JT. Remodeling and homeostasis of the extracellular matrix: implications for fibrotic diseases and cancer. Dis Model Mech. 2011;4(2):165–78. doi: 10.1242/dmm.004077 21324931 PMC3046088

[6] Walker CA, Spinale FG. The structure and function of the cardiac myocyte: a review of fundamental concepts. J Thorac Cardiovasc Surg. 1999;118(2):375–82. doi: 10.1016/S0022-5223(99)70233-3 10425017

[7] Hughes CJR, Jacobs JR. Dissecting the Role of the Extracellular Matrix in Heart Disease: Lessons from the Drosophila Genetic Model. Vet Sci. 2017;4(2):24. doi: 10.3390/vetsci4020024 29056683 PMC5606597

[8] Hughes CJR, Turner S, Andrews RM, Vitkin A, Jacobs JR. Matrix metalloproteinases regulate ECM accumulation but not larval heart growth in Drosophila melanogaster. J Mol Cell Cardiol. 2020;140:42–55. doi: 10.1016/j.yjmcc.2020.02.008 32105665

[9] Mouw JK, Ou G, Weaver VM. Extracellular matrix assembly: a multiscale deconstruction. Nat Rev Mol Cell Biol. 2014;15(12):771–85. doi: 10.1038/nrm3902 25370693 PMC4682873

[10] Radisky ES, Radisky DC. Matrix metalloproteinases as breast cancer drivers and therapeutic targets. Front Biosci (Landmark Ed). 2015;20(7):1144–63. doi: 10.2741/4364 25961550 PMC4516284

[11] Zhou P, Yang C, Zhang S, Ke Z-X, Chen D-X, Li Y-Q, et al. The Imbalance of MMP-2/TIMP-2 and MMP-9/TIMP-1 Contributes to Collagen Deposition Disorder in Diabetic Non-Injured Skin. Front Endocrinol (Lausanne). 2021;12:734485. doi: 10.3389/fendo.2021.734485 34777244 PMC8579102

[12] Meschiari CA, Ero OK, Pan H, Finkel T, Lindsey ML. The impact of aging on cardiac extracellular matrix. Geroscience. 2017;39(1):7–18. doi: 10.1007/s11357-017-9959-9 28299638 PMC5352584

[13] Philp CJ, Siebeke I, Clements D, Miller S, Habgood A, John AE, et al. Extracellular Matrix Cross-Linking Enhances Fibroblast Growth and Protects against Matrix Proteolysis in Lung Fibrosis. Am J Respir Cell Mol Biol. 2018;58(5):594–603. doi: 10.1165/rcmb.2016-0379OC 29053339

[14] Pehrsson M, Mortensen JH, Manon-Jensen T, Bay-Jensen A-C, Karsdal MA, Davies MJ. Enzymatic cross-linking of collagens in organ fibrosis – resolution and assessment. Expert Review of Molecular Diagnostics. 2021;21(10):1049–64. doi: 10.1080/14737159.2021.1962711 34330194

[15] El Hajj EC, El Hajj MC, Ninh VK, Gardner JD. Inhibitor of lysyl oxidase improves cardiac function and the collagen/MMP profile in response to volume overload. Am J Physiol Heart Circ Physiol. 2018;315(3):H463–73. doi: 10.1152/ajpheart.00086.2018 29775412 PMC6172641

[16] Cavalera M, Wang J, Frangogiannis NG. Obesity, metabolic dysfunction, and cardiac fibrosis: pathophysiological pathways, molecular mechanisms, and therapeutic opportunities. Transl Res. 2014;164(4):323–35. doi: 10.1016/j.trsl.2014.05.001 24880146 PMC4180761

[17] Pastor-Pareja JC. Atypical basement membranes and basement membrane diversity - what is normal anyway?. J Cell Sci. 2020;133(8):jcs241794. doi: 10.1242/jcs.241794 32317312

[18] Rotstein B, Paululat A. On the Morphology of the Drosophila Heart. J Cardiovasc Dev Dis. 2016;3(2):15. doi: 10.3390/jcdd3020015 29367564 PMC5715677

[19] Chartier A, Zaffran S, Astier M, Sémériva M, Gratecos D. Pericardin, aDrosophilatype IV collagen-like protein is involved in the morphogenesis and maintenance of the heart epithelium during dorsal ectoderm closure. Development. 2002;129(13):3241–53. doi: 10.1242/dev.129.13.3241 12070098

[20] Sessions AO, Kaushik G, Parker S, Raedschelders K, Bodmer R, Van Eyk JE, et al. Extracellular matrix downregulation in the Drosophila heart preserves contractile function and improves lifespan. Matrix Biol. 2017;62:15–27. doi: 10.1016/j.matbio.2016.10.008 27793636 PMC5405015

[21] Drechsler M, Schmidt AC, Meyer H, Paululat A. The conserved ADAMTS-like protein lonely heart mediates matrix formation and cardiac tissue integrity. PLoS Genet. 2013;9(7):e1003616. doi: 10.1371/journal.pgen.1003616 23874219 PMC3708815

[22] Birse RT, Choi J, Reardon K, Rodriguez J, Graham S, Diop S, et al. High-fat-diet-induced obesity and heart dysfunction are regulated by the TOR pathway in Drosophila. Cell Metab. 2010;12(5):533–44. doi: 10.1016/j.cmet.2010.09.014 21035763 PMC3026640

[23] Diop SB, Birse RT, Bodmer R. High Fat Diet Feeding and High Throughput Triacylglyceride Assay in <em>Drosophila Melanogaster</em>. JoVE. 2017;(127):56029. doi: 10.3791/56029 28930984 PMC5752231

[24] Diop SB, Bodmer R. Drosophila as a model to study the genetic mechanisms of obesity-associated heart dysfunction. J Cell Mol Med. 2012;16(5):966–71. doi: 10.1111/j.1582-4934.2012.01522.x 22303936 PMC3454526

[25] Guida MC, Birse RT, Dall’Agnese A, Toto PC, Diop SB, Mai A, et al. Intergenerational inheritance of high fat diet-induced cardiac lipotoxicity in Drosophila. Nat Commun. 2019;10(1). doi: 10.1038/s41467-018-08128-3 PMC6331650 30643137

[26] Rewitz KF, Yamanaka N, O’Connor MB. Developmental checkpoints and feedback circuits time insect maturation. Curr Top Dev Biol. 2013;103:1–33. doi: 10.1016/B978-0-12-385979-2.00001-0 23347514 PMC4060521

[27] Hinderer S, Schenke-Layland K. Cardiac fibrosis - A short review of causes and therapeutic strategies. Adv Drug Deliv Rev. 2019;146:77–82. doi: 10.1016/j.addr.2019.05.011 31158407

[28] Walls SM, Chatfield DA, Ocorr K, Harris GL, Bodmer R. Systemic and heart autonomous effects of sphingosine Δ4 desaturase deficiency in lipotoxic cardiac pathophysiology. Dis Model Mech. 2020;13(8):dmm043083. doi: 10.1242/dmm.043083 32641420 PMC7438009

[29] Na J, Musselman LP, Pendse J, Baranski TJ, Bodmer R, Ocorr K, et al. A Drosophila Model of High Sugar Diet-Induced Cardiomyopathy. PLoS Genet. 2013;9(1):e1003175. doi: 10.1371/journal.pgen.1003175 PMC3542070 23326243

[30] Palanker Musselman L, Fink JL, Narzinski K, Ramachandran PV, Sukumar Hathiramani S, Cagan RL, et al. A high-sugar diet produces obesity and insulin resistance in wild-type *Drosophila*. Disease Models & Mechanisms. 2011;4(6):842–9. doi: 10.1242/dmm.007948 21719444 PMC3209653

[31] Buszczak M, Paterno S, Lighthouse D, Bachman J, Planck J, Owen S, et al. The carnegie protein trap library: a versatile tool for Drosophila developmental studies. Genetics. 2007;175(3):1505–31. doi: 10.1534/genetics.106.065961 17194782 PMC1840051

[32] Zeng J, Huynh N, Phelps B, King-Jones K. Snail synchronizes endocycling in a TOR-dependent manner to coordinate entry and escape from endoreplication pausing during the Drosophila critical weight checkpoint. PLoS Biol. 2020;18(2):e3000609. doi: 10.1371/journal.pbio.3000609 32097403 PMC7041797

[33] Carbone S, Mauro AG, Mezzaroma E, Kraskauskas D, Marchetti C, Buzzetti R, et al. A high-sugar and high-fat diet impairs cardiac systolic and diastolic function in mice. Int J Cardiol. 2015;198:66–9. doi: 10.1016/j.ijcard.2015.06.136 26151718

[34] Martins Matias A, Murucci Coelho P, Bermond Marques V, dos Santos L, Monteiro de Assis ALE, Valentim Nogueira B, et al. Hypercaloric diet models do not develop heart failure, but the excess sucrose promotes contractility dysfunction. PLoS ONE. 2020;15(2):e0228860. doi: 10.1371/journal.pone.0228860 PMC7006916 32032383

[35] Hardy CM, Birse RT, Wolf MJ, Yu L, Bodmer R, Gibbs AG. Obesity-associated cardiac dysfunction in starvation-selected Drosophila melanogaster. Am J Physiol Regul Integr Comp Physiol. 2015;309(6):R658-67. doi: 10.1152/ajpregu.00160.2015 26136533 PMC4591367

[36] Fan D, Takawale A, Lee J, Kassiri Z. Cardiac fibroblasts, fibrosis and extracellular matrix remodeling in heart disease. Fibrogenesis Tissue Repair. 2012;5(1):15. doi: 10.1186/1755-1536-5-15 22943504 PMC3464725

[37] Berk BC, Fujiwara K, Lehoux S. ECM remodeling in hypertensive heart disease. J Clin Invest. 2007;117(3):568–75. doi: 10.1172/jci31044 17332884 PMC1804378

[38] Garrett SM, Hsu E, Thomas JM, Pilewski JM, Feghali-Bostwick C. Insulin-like growth factor (IGF)-II- mediated fibrosis in pathogenic lung conditions. PLoS ONE. 2019;14(11):e0225422. doi: 10.1371/journal.pone.0225422 PMC6876936 31765403

[39] Tian G, Luo C, Liu L. Epicardial adipose tissie-derived leptin induce mmps/timps imbalance and promote cardiac fibrosis through jak2/ros/na/k-atpase/erk1/2 signaling pathway in high fat diet-induced obese rats. Journal of the American College of Cardiology. 2022;79(9):1544. doi: 10.1016/s0735-1097(22)02535-935422251

[40] Alpert MA, Omran J, Bostick BP. Effects of Obesity on Cardiovascular Hemodynamics, Cardiac Morphology, and Ventricular Function. Curr Obes Rep. 2016;5(4):424–34. doi: 10.1007/s13679-016-0235-6 27744513

[41] Nishida K, Otsu K. Inflammation and metabolic cardiomyopathy. Cardiovasc Res. 2017;113(4):389–98. doi: 10.1093/cvr/cvx012 28395010

[42] Hartley PS, Motamedchaboki K, Bodmer R, Ocorr K. SPARC-Dependent Cardiomyopathy in Drosophila. Circ Cardiovasc Genet. 2016;9(2):119–29. doi: 10.1161/CIRCGENETICS.115.001254 26839388 PMC4838489

[43] DeLeon-Pennell KY, Meschiari CA, Jung M, Lindsey ML. Matrix Metalloproteinases in Myocardial Infarction and Heart Failure. Prog Mol Biol Transl Sci. 2017;147:75–100. doi: 10.1016/bs.pmbts.2017.02.001 28413032 PMC5576003

[44] McCurdy SM, Dai Q, Zhang J, Zamilpa R, Ramirez TA, Dayah T, et al. SPARC mediates early extracellular matrix remodeling following myocardial infarction. Am J Physiol Heart Circ Physiol. 2011;301(2):H497-505. doi: 10.1152/ajpheart.01070.2010 21602472 PMC3154667

[45] Wat LW, Chao C, Bartlett R, Buchanan JL, Millington JW, Chih HJ, et al. A role for triglyceride lipase brummer in the regulation of sex differences in Drosophila fat storage and breakdown. PLoS Biol. 2020;18(1):e3000595. doi: 10.1371/journal.pbio.3000595 PMC6994176 31961851

[46] Brent JR, Werner KM, McCabe BD. Drosophila Larval NMJ Dissection. JoVE. 2009;(24):1107. doi: 10.3791/1107 19229190 PMC2762896

[47] Abràmoff DMD, Magalhaes PJ, Ram S. Image Processing with ImageJ. Biophotonics Int. 2004.

[48] Gómez IM, Rodríguez MA, Santalla M, Kassis G, Colman Lerner JE, Aranda JO, et al. Inhalation of marijuana affects Drosophila heart function. Biol Open. 2019;8(8):bio044081. doi: 10.1242/bio.044081 31324618 PMC6737967

