# Supplementary figures and images for: Cardiac function and extracellular matrix morphology are altered by chronic high fat diet in Drosophila larvae
Source: PLoS One. 2025 Aug 22;20(8):e0330487. doi: 10.1371/journal.pone.0330487 (PMC12373202; doi:10.1371/journal.pone.0330487)

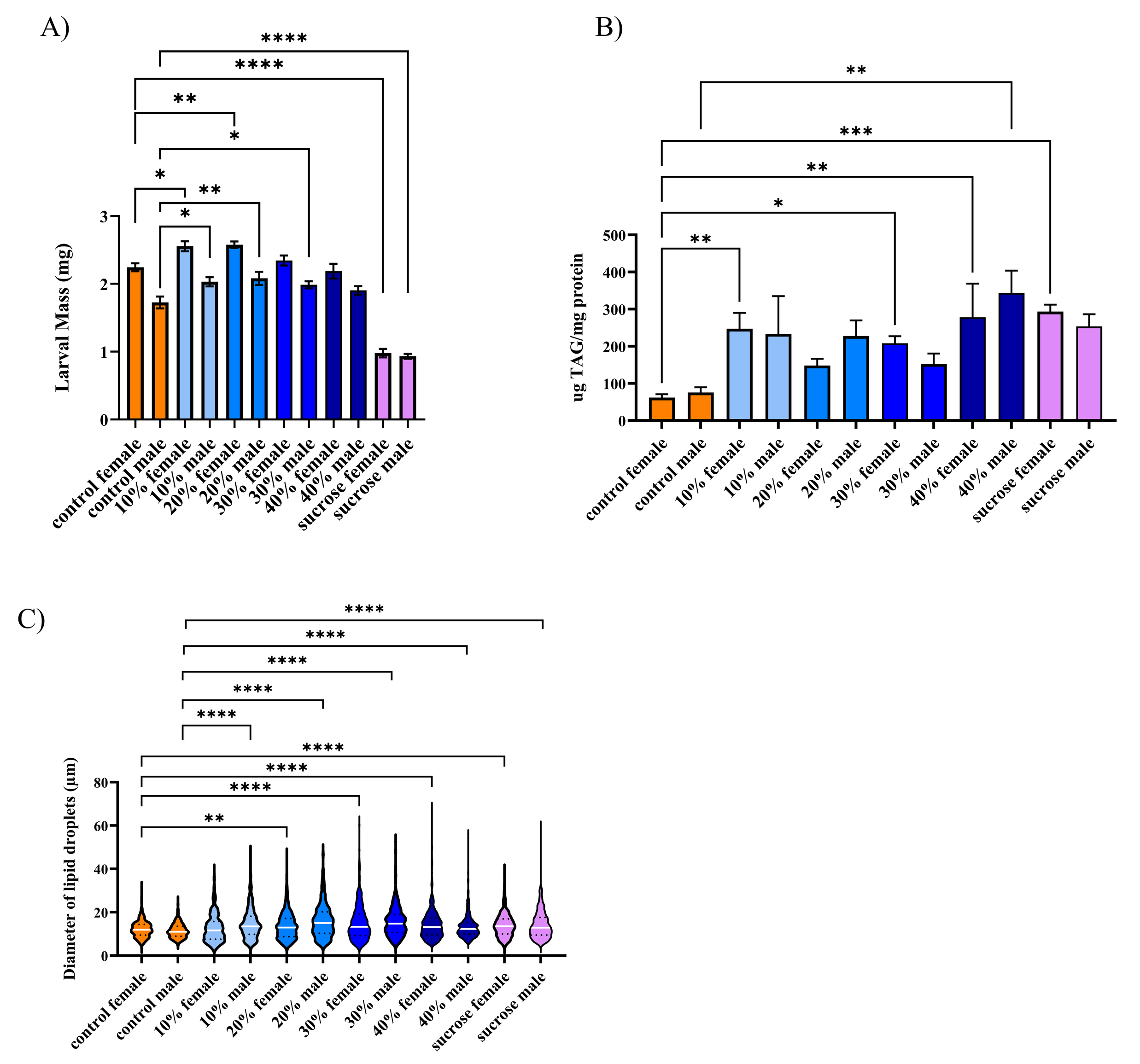

Supplement: S1 Fig — Figs 1A, B, and D organized to show female and male data beside each other. Error bars are SEM. * = p < 0.05, ** = p < 0.01, *** = p < 0.001, **** = p < 0.0001. If no p value is indicated, comparison is not statistically significant. (TIF) [file pone.0330487.s001.tif]

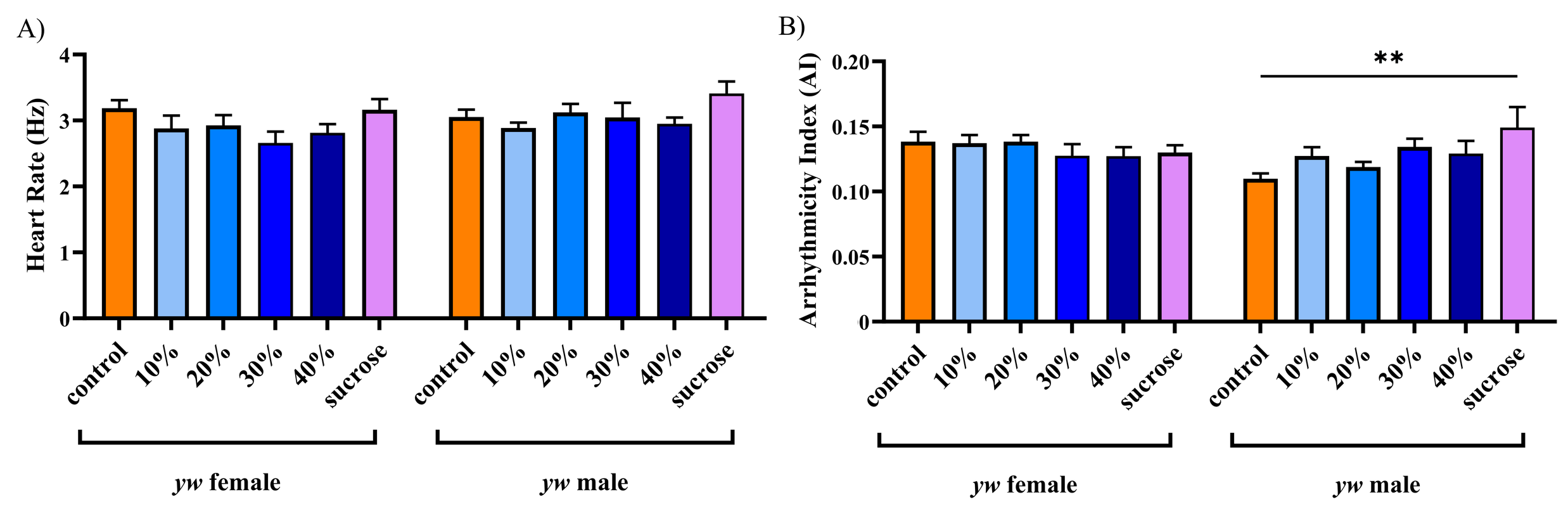

Supplement: S2 Fig — Heart rate was not significantly different with any dietary treatment (A). Arrhythmicity index was unaffected in all treatments except for high sucrose diet males (B). Error bars are SEM. ** = p < 0.01. (TIF) [file pone.0330487.s002.tif]
